# Economic evaluation of a childhood obesity prevention programme for children: Results from the WAVES cluster randomised controlled trial conducted in schools

**DOI:** 10.1371/journal.pone.0219500

**Published:** 2019-07-10

**Authors:** Alastair Canaway, Emma Frew, Emma Lancashire, Miranda Pallan, Karla Hemming, Peymane Adab, on behalf of the WAVES trial investigators

**Affiliations:** Institute of Applied Health Research, University of Birmingham, Birmingham, United Kingdom; University College London, UNITED KINGDOM

## Abstract

**Background:**

Childhood obesity is a serious public health challenge and schools have been identified as an ideal place to implement prevention interventions. The aim of this study was to measure the cost-effectiveness of a multi-faceted school-based obesity prevention intervention targeting children aged 6–7 years when compared to ‘usual activities’.

**Methods:**

A cluster randomised controlled trial in 54 schools across the West Midlands (UK) was conducted. The 12-month intervention aimed to increase physical activity by 30 minutes per day and encourage healthy eating. Costs were captured from a public sector perspective and utility-based health related outcomes measured using the CHU-9D. Multiple imputation using chained equations was used to address missing data. The cost effectiveness was measured at 30 months from baseline using a hierarchical net-benefit regression framework, that controlled for clustering and prespecified covariates. Any uncertainty in the results was characterised using cost-effectiveness acceptability curves.

**Results:**

At 30 months, the total adjusted incremental mean cost of the intervention was £155 (95% confidence interval [CI]: £139, £171), and the incremental mean QALYs gained was 0.006 (95% CI: -0.024, 0.036), per child. The incremental cost-effectiveness at 30 months was £26,815 per QALY and using a standard willingness to pay threshold of £30,000 per QALY, there was a 52% chance that the intervention was cost-effective.

**Conclusions:**

The cost-effectiveness of the school-based WAVES intervention was subject to substantial uncertainty. We therefore recommend more research to explore obesity prevention within schools as part of a wider systems approach to obesity prevention.

**Trial registration:**

This paper uses data collected by the WAVES trial: Controlled trials ISRCTN97000586 (registered May 2010).

## Introduction

Childhood obesity is one of the most serious global public health challenges of modern times. Recent reports state that it has increased ten-fold since 1975, affecting 41 million children under the age of 5 years[1]. Children with obesity are at risk of adverse physical health, emotional, and social consequences [2, 3] and, if overweight in childhood, have a high risk of being overweight in adulthood[4]. There are considerable economic costs associated with obesity in the form of increased health care spending, and productivity costs[4].

In the UK, obesity prevalence doubles during the primary school years from reception age (age 4–5 years) to Year 6 (age 10–11 years). The primary school age period is therefore a critical window of opportunity for preventive intervention. The West Midlands ActiVe lifestyle and healthy Eating in School children (WAVES) cluster-randomised controlled trial, completed in 2016, evaluated a multi-component intervention, targeting both the school and family environment. A small difference in BMI z-scores between intervention and control groups was found, in favour of the intervention, although this difference was not statistically significant [5]. Given the possibility of a small beneficial effect of the intervention, it is important to estimate the potential cost-effectiveness of this intervention. These cost-effectiveness results are important to decision makers where the focus is on the ratio of cost differences to effect differences as in some circumstances even a small change in effect can be cost-effective when considered against competing claims for health care resources [6]. Increasingly, economic evidence is being used to inform decision making in many countries [7–10]. In particular, it offers a robust approach to determine value for money when the health gains [or losses] are measured using a standard generic quality of life measure and are assessed against any additional costs [or savings]. The results of such a cost-effectiveness analysis will therefore help to inform decisions on whether to implement multi-component interventions such as WAVES within primary schools.

The purpose of this paper is to report on the cost-effectiveness analysis conducted alongside the WAVES cluster-randomised controlled trial. The study was conducted in the UK, within primary schools located in the West Midlands. The evaluation adopted a public sector perspective, comparing the cost-effectiveness of schools implementing the WAVES intervention in comparison to schools that continued with usual healthy lifestyle activities. The clinical effectiveness results of the trial have been previously reported[5].

## Methods

Full details of the WAVES study design and intervention can be found in the main clinical trial paper [5] and in the WAVES protocol paper[11]. The trial was a school based, cluster-randomised, controlled trial. Participating state primary schools were located within the West Midlands, UK. Schools were randomised to receive either the intervention or to continue with ongoing health-related activities (the control arm). The target group was Year 2 children (aged 6–7 years), and the intervention, delivered over 12 months, comprised four components: a school based physical activity component; a dietary activity component; a 6-week programme delivered by an iconic sporting institution to encourage healthy eating and physical activity; and family signposting to activities outside of school. Ethical approval for the study was granted by the NHS Research Ethics Service (NHS REC no.10/H1202/69).

The within-trial economic evaluation was undertaken from a public sector perspective. It considered costs and effects from baseline to 18 months after the end of the intervention (30 months from baseline), although results at 3 months after the intervention (15 months from baseline) were also reported. A cost utility analysis was performed that considered difference in cost alongside difference in effect measured in Quality Adjusted Life Years (QALYs) using the CHU-9D quality of life questionnaire [11,12], an instrument validated for use in a paediatric population[12]. All prices were adjusted to 2014 prices and any costs/QALYs that accrued after the first year of the trial were discounted at 3.5% per year[8].

### Resource use and costs

Two categories of resource use were collected: the resource use associated with the development of the intervention, and the resource use associated with the delivery of the intervention. Only the costs incurred for the delivery of the intervention were included in the main economic evaluation, however details of the intervention development costs are available within the supplementary material (S1 Table). All resource use costs were collected and presented at the school class level.

The dietary activity component involved three workshops delivered at school, each focusing on one main meal: breakfast, lunch or dinner. Materials and non-perishable food items were purchased by the research team and delivered to schools. Cost for these items was carefully recorded as were the costs incurred for their delivery to schools. School staff were asked to record using a logbook the costs for any additional items purchased. This enabled the cost per class to be estimated.

Each school was invited to choose two out of four physical activity packages to help deliver an additional 30 minutes of physical activity during the school day. Depending on which package was selected, these either took place within classroom sessions, or during break times. The resource use associated with this aspect of the intervention was captured through school staff-completed logbooks.

For the 6-week programme delivered by the iconic sporting institution, each school had: two visits to Birmingham Aston Villa Football club to attend ‘Villa Vitality days’ which aimed to convey knowledge and skills related to healthy eating and physical activity; one school-based Villa-Vitality session (run by football club staff in school; a variety of home challenges and a class project which aimed to teach the children about the benefits of physical activity and nutrition. In terms of resource use, the Villa Vitality package was purchased at a fixed cost. Any time school staff spent on the class projects and home challenges was captured through staff logbooks.

The final intervention component involved the development of information sheets for each school to signpost local healthy activities outside of school. The research team were responsible for developing, updating and delivering these materials to the schools. Time spent by staff on this activity was recorded using an electronic log. Purchase orders and invoices were kept to capture the resource required to produce the leaflets.

For staff time, unit costs were obtained from publicly available sources including the Department of Education[13], University staff pay scales [14] and Office of National Statistics (ONS) earning surveys[15]. Where relevant, mid-scale salary points were used to assign cost to school and research staff time. For the cooking workshops, the unit costs for the cooking materials were based on the purchase price of each item. Any fixed costs associated with the physical activity packages were recorded by the research trial team and receipts retained. For the travel costs associated with the delivery of materials to schools, an assumed rate of £0.45 was applied based on HMRC guidance[16].

For our base case analysis (the main economic evaluation) we assumed an average class size of 30 children as all resource use data associated with the delivery of the intervention were collected at the school level. As the control arm in this study had no intervention, no costs were associated with this arm.

### Outcome measures

QALYs were calculated from the generic preference-based Child Health Utility 9-Dimension (CHU-9D) questionnaire[17]. The CHU-9D was interview-administered at baseline, follow up 1 (3-months after the intervention), and follow up 2 (18-months after the intervention). The CHU-9D questionnaire generated a health-related quality of life score for each individual child expressed as a utility value. The QALYs were then constructed by combining these utility estimates with time, using the area under the curve method[18].

### Missing data

Missing values were imputed using multiple imputation by chained equations and this was done separately for costs and outcomes as the reasons for missing data varied. Missing values for outcomes incorporated both individual-level covariates (gender, ethnicity, deprivation, baseline total energy consumption, baseline physical activity energy expenditure) and cluster covariates (school size, school ethnicity, free-school eligibility). Missing values for costs incorporated only the prespecified cluster covariates [19]. This was done using REALCOM-IMPUTE software in conjunction with STATA 13. Thirty imputations were conducted, resulting in 30 complete data sets. Rubin’s rule [20], which incorporates uncertainty around the predicted values, was used to calculate pooled estimates of the mean costs and QALYs, as well as the confidence intervals.

### Cost-effectiveness analysis

To begin, costs and outcomes were analysed separately using a multilevel regression framework to control for prespecified covariates and cluster randomisation. The prespecified covariates that were adjusted for included cluster level covariates used in the randomisation (school size, proportion of children eligible for free school meals, ethnicity), and child-level covariates (gender, baseline CHU9D score, ethnicity, deprivation, baseline total energy consumption and baseline physical activity energy expenditure).

To estimate the cost-effectiveness, the difference in costs were then compared to the difference in outcomes for the intervention compared to the control arm. To account for both the correlation between costs and outcomes and the cluster-design of the trial, the net-benefit regression (NBR) framework was applied [21–24]. The net benefit (*nb*) is defined for each participant in the trial as follows:

nb=e.λ−c

where *e* is the observed effect, *λ* is the value that society is willing to pay for an effect-gain, and *c* is the estimated cost for each participant. The incremental net benefit (*INB*) is the difference between the expected net benefit of the WAVES intervention (*nb*_*WAVES*_) and the expected net benefit of the control (*nb*_*control*_). The nb is calculated for every participant and then used as a dependent variable within a standard regression framework as follows:

nb=∝+δTrialArm+,…,+ε

The coefficient of the explanatory variable (*TrialArm*) is then used to estimate the *INB* as estimates of *δ* are estimates of (*nb*_*WAVES*_−*nb*_*control*_), see Hoch and Dewa, 2014 for further explanation[22]. The confidence intervals for the coefficient *δ* are the confidence intervals for the *INB*. A separate regression was run assuming different willingness to pay values (*λ*) for a QALY gain, to estimate the corresponding *INB*, and confidence intervals. This process assesses how sensitive the cost-effectiveness recommendations are to the willingness to pay assumptions.

If the INB >0 at a given level of *λ* then the intervention is considered cost-effective. By applying the NBR framework, the cluster randomisation and covariates were also accounted for using the same prespecified variables as that used to independently estimate the cost and effect differences. This was done using multi-level modelling that modelled the individual children as the first level, and then the clusters as the second level.

The results of the NBR framework were presented in the form of a cost-effectiveness acceptability curve (CEAC) which showed the probability of the intervention being cost-effective at different values of *λ*. In the UK, an intervention is considered good value for money if the cost per QALY value is less than the threshold value of £30,000 per QALY-gain[25].

### Secondary analysis

The cost-effectiveness was also assessed in terms of ‘cost per obesity case prevented’. Cases of interest were defined as children who were not obese at baseline but then had developed obesity 18 months after the intervention. Height and weight were measured by trained researchers at school using standardised protocols. Weight status groups were calculated using the UK 1990 BMI cut-offs as recommended by NICE [26, 27]. The prior assumption was, if the WAVES intervention was effective, then fewer children would be transitioning from a non-obese weight category to an obese weight category in the intervention arm, compared to the control arm. To explore this a transition dummy variable was created to represent if the child’s weight status changed to obese during the trial period. A multilevel logit model including a dummy variable for the intervention was applied to assess the intervention effect, while controlling for cluster randomisation and prespecified covariates. This was done using STATA 13 and the ‘melogit’ command, with the school variable being the level 2 identifier.

### Sensitivity Analyses

Three sensitivity analyses were conducted to examine how robust the results were to any assumptions made. First, an alternative method for analysis was adopted. This involved using a fixed effect multiple imputation approach with seemingly unrelated regressions for estimation. In the second sensitivity analysis, the incremental net benefit was re-calculated with the costs of setting up the intervention included. The final sensitivity analysis inflates the cost to i) match the average class size in the study and ii) attach the costs to only those children who consented for participation. All data were analysed using the intention to treat principle in STATA version 13.0[28].

## Results

The WAVES trial recruited 54 schools and obtained parental consent for participation within the trial from 1,467 children. School characteristics between the intervention and the control schools were broadly balanced, however there were baseline imbalances in some of the characteristics at the participant level, as detailed in Table 1 and reported in full within the main clinical-effectiveness paper[5]. The average age of the sample was 6.3 (SD = 0.3) years at baseline, 51% were male. The sample was mostly from a white British ethnic origin, 31% were South Asian and 8% were Black African Caribbean. The sample was predominantly from a deprived background with 55% falling into the most deprived IMD quintile.

**Table 1:** Baseline characteristics of pupils in the WAVES study by trial arm.

Characteristics	Intervention arm	Control arm	Total
**Age:**	n = 622	n = 735	n = 1397
Mean (SD) age (years); not known	6.3 (0.3); 27	6.3(0.3); 43	6.3(0.3); 70
**Sex:**	n = 689	n = 778	n = 1467
Male	339 (49.2)	410 (52.7)	749 (51.1)
Female	350 (50.8)	368 (47.3)	718 (48.9)
**Ethnicity:**	n = 676	n = 775	n = 1451
White British	297(43.9)	361(46.6)	658(45.3)
South Asian	221(32.7)	222(28.6)	443(30.5)
Black African- Caribbean	62(9.2)	53(6.8)	115(7.9)
Other	96(14.2)	139(17.9)	235(16.2)
Not known	13	3	16
Deprivation*:	n = 670	n = 769	n = 1439
1 (most deprived)	392(58.5)	398(51.8)	790(54.9)
2	120(17.9)	154(20.0)	274(19.0)
3	72(10.7)	74(9.6)	146(10.1)
4	65(9.7)	54(7.0)	119(8.3)
5 (least deprived)	21(3.1)	89(11.6)	110(7.6)
**BMI z-score:**	n = 660	n = 732	n = 1392
Mean (SD) BMI z-score; not known	0.23(1.2); 29	0.15(1.2);46	0.19(1.2);75
Weight status**	n = 660	n = 732	n = 1392
Underweight (£ 2nd centile)	20 (3.0)	20 (2.7)	40 (2.9)
Healthy weight (>2nd and <85th centiles)	495 (75.0)	562 (76.8)	1057 (75.9)
Overweight (^3^85th and <95th centiles)	61 (9.2)	63 (8.6)	124 (8.9)
Obese (^3^95th centile)	84 (12.7)	87 (11.9)	171 (12.3)
Not known	29	46	75

*Index of multiple deprivation

** Based on UK 1990 reference centile curves and applying cut-offs used for population monitoring.

### Health outcomes

Table 2 shows the health-related quality of life utility scores at each follow up point by treatment arm. Eighteen months after the intervention, children who received the intervention experienced 2.171 QALYs, compared to 2.141 QALYs for children with no intervention. After adjustments for clustering, baseline differences and the pre-specified covariates using multi-level regression, the mean difference in QALYs between the two arms at follow up 2 was 0.006 (95% CI -0.024 to 0.036; p = 0.701).

**Table 2:** Mean utility scores and QALYs per child by treatment arm.

	n	Intervention arm, mean (95% CI)	Control arm, mean (95% CI)
**Utility score**			
**Baseline**	1350	0.836 (0.826 to 0.846)	0.816 (0.805 to 0.827)
**FU 1**	1215	0.868 (0.868 to 0.877)	0.858 (0.850 to 0.866)
**FU 2**	1128	0.893 (0.884 to 0.901)	0.898 (0.891 to 0.905)
**QALY Differences**	**Mean Difference** **(95% CI)**	**p-value**	
**Unadjusted**			
**FU1**	0.018 (0.005 to 0.031)	0.006	
**FU2**	0.019 (-0.003 to 0.041)	0.097	
**Adjusted** *			
**FU 1**	0.005 (-0.008 to 0.018)	0.429	
**FU 2**	0.006 (-0.024 to 0.036)	0.701	

*adjusted for baseline cluster-level covariates (school size, proportion of children eligible for free school meals, ethnicity) and baseline child-level covariates (gender, baseline CHU9D score, ethnicity, deprivation, baseline total energy consumption and baseline physical activity energy expenditure).

### Resource use and costs

The mean resource use and cost per school class for the delivery of the intervention were estimated (Table 3). The total mean cost of the intervention per class and per child was £4,597 and £153, respectively. These costs reflect all four components of the intervention, with the greatest cost component being the ‘villa vitality’ package. After adjusting for clustering, and the covariates, the incremental cost of the intervention compared to the control arm was £155.53 per child (95% CI £139.97, £171.09).

**Table 3:** Mean resource use and costs per class to deliver the 12-month intervention.

Resource Use Item	Unit cost	Resource Use per Class	Mean cost per class (£)
**Signposting:**	** **		
Research associate (staff time–hours)	15.17 (per hour)	6.325	95.99
Study administrator (staff time–hours)	11.66 (per hour)	0.938	10.94
Generic printing (sheets)	22p (per sheet)	38.75	8.88
School printing (sheets)	37p (per sheet)	38.81	14.38
Delivery to schools	n/a	n/a	2.94
**Villa Vitality:**			
Fixed cost of package			1,979.66
Teacher time (hours)	20.09	31.61	635.1
Teacher assistant time (hours)	9.04	28.84	260.74
**Cooking classes:**	** **		
Teacher time (hours)	20.09	4.18	83.92
Teacher assistant time (hours)	9.04	0.85	7.68
**Cooking workshops:**			
Teacher time (hours)	20.08	5.94	119.31
Teacher assistant time (hours)	9.04	0.74	6.69
Other staff helpers (hours)	9.05	6.33	57.31
**Purchasing/packing materials:**			
Research Fellow (hours)	18.83	0.3	5.56
Research Associate (hours)	16.47	0.9	14.83
Admin Staff (hours)	11.7	0.1	1.17
**Printing materials**	n/a		100.09
**Cooking materials**			
Breakfast	n/a		25.47
Lunch	n/a		8.5
Dinner	n/a		6.99
**Delivery of materials**			
Staff time (hours)	16.46	2.54	41.82
Travel cost	** **		33.66
**Physical Activity:**	** **		
Teacher time (hours)	20.3	47.37	961.92
Teacher assistant time (hours)	9.09	2.15	19.56
Learning and Teaching support staff (hours)	7.32	12.87	94.26
**Total mean cost per school**			7072.88
**Total mean cost per class**			4597.37
**Total mean cost per consented child**			263.46
**Total mean cost per child with average class size (28.45)**			161.59
Total mean cost per child assuming a class size of 30*			153.2

*Cost estimate used for main analyses

### Cost-utility analysis

The difference in costs was combined with the difference in effects to form an ICER. At follow up two, 18 months after the end of the intervention, the cost per additional QALY for the intervention compared to the control was £26,815 per QALY. This means that each additional QALY gained from the intervention costs £26,815.

**Fig 1:**
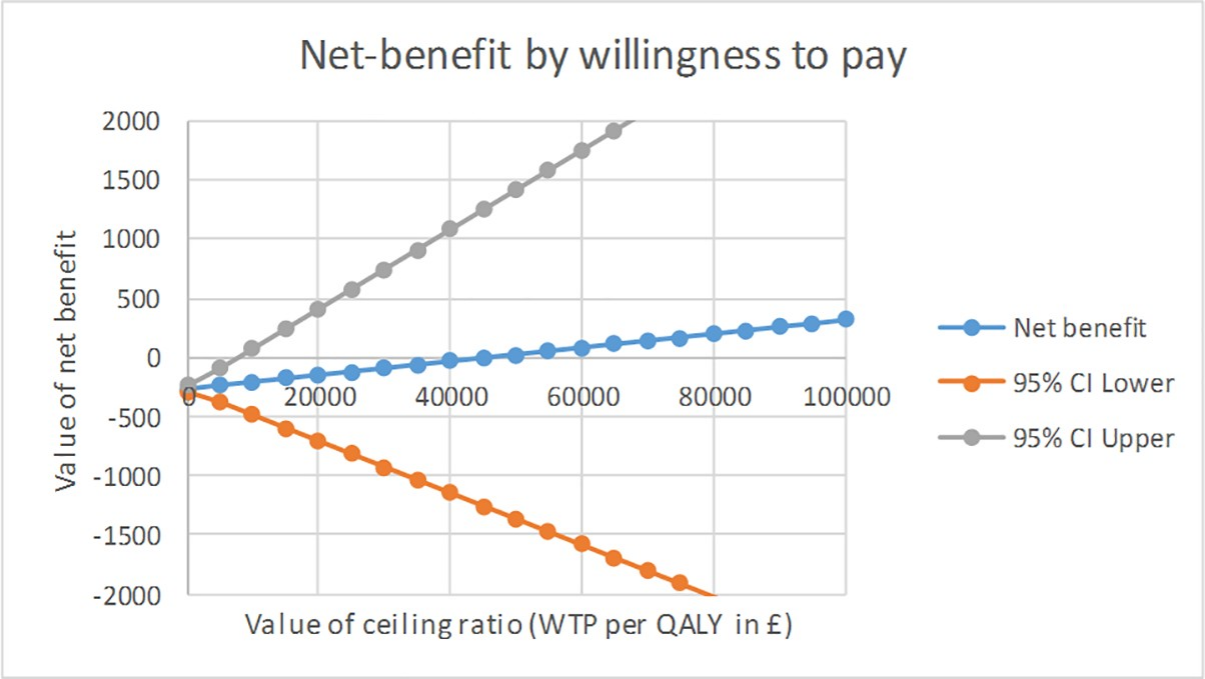
Net-benefit by threshold willingness to pay for an additional QALY.

Using the net benefit framework, the level of uncertainty around the ICER was graphically presented (Fig 1) adjusting for the pre-specified covariates and clustering.

**Fig 2:**
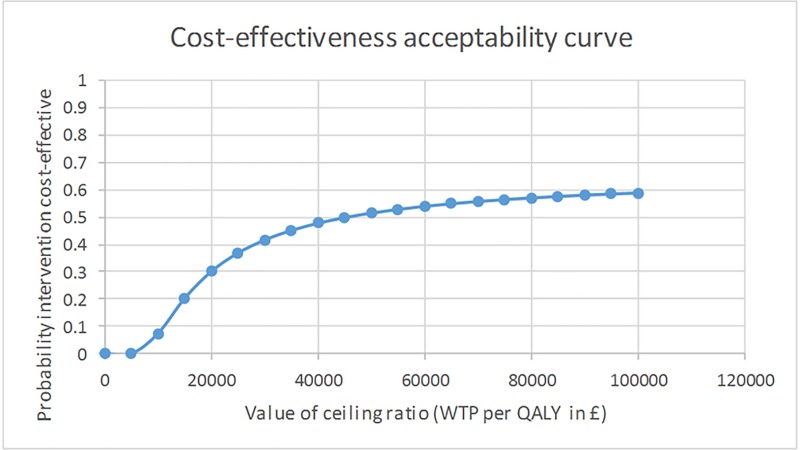
Cost Effectiveness Acceptability Curve.

The CEAC presents the probability that the WAVES intervention is cost effective at varying threshold levels of willingness to pay (Fig 2). At the commonly applied UK threshold of £30,000 per QALY, the CEAC showed that the probability of the WAVES intervention being cost-effective was only 52%, and even at high levels of willingness to pay for an additional QALY-gain, this probability of cost-effectiveness did not rise above 62%.

### Sensitivity analyses

The first sensitivity analysis used an alternative approach to imputing and analysing the data (seemingly unrelated regressions). This had minimal effects on the cost (£153) however incremental QALYs were reduced to 0.0033. This had the result of increasing the ICER to £46,363 per QALY. The second sensitivity analysis included the intervention set up and implementation costs, which led to an increase in mean cost per child to £199, which resulted in an ICER of £34,000 per additional QALY. With all sensitivity analyses there remained a high level of uncertainty of less than 63% probability of cost-effectiveness even when the threshold willingness to pay value was set at £100,000. The third sensitivity analysis adjusted the costs upwards to first match the average class size and then for only the consented children. This increased the ICER to £28,265 and £46,083 respectively.

Within the trial, proportionally more children within the intervention arm (10%) compared to the control arm (7%) transitioned from a baseline healthy weight to a state defined as having obesity at follow up two. A multilevel logit model from the main trial analysis [29] demonstrated that the odds ratio associated with children in the intervention arm transitioning to living with obesity at follow up two, was 1.17 (0.66, 2.09), when controlling for pre-specified covariates. Although this was not a statistically significant difference, this estimated effect in favour of the control arm made the planned economic analysis for estimating the cost per ‘case of childhood obesity prevented’ not possible, that is, the control arm dominated the intervention arm.

## Discussion

The WAVES study was unable to provide clear evidence that a childhood obesity prevention programme delivered through schools was effective at preventing childhood obesity[5]. In this economic evaluation, at 18 months post intervention, the WAVES intervention was found to be slightly more effective in QALY terms but also more expensive when compared to the control arm. The ICER was estimated at £26,815 per QALY which is within the range of what would commonly be regarded as a cost-effective use of public resources within the UK, however, there was a high level of uncertainty as demonstrated by the net-benefit equation and the corresponding CEAC. The probability (adjusted for covariates and clustering) of the WAVES intervention achieving an additional QALY when the threshold willingness to pay was set at £30,000, was only 52%. This result reflects the high level of uncertainty found when estimating the incremental QALY gain evidenced by the confidence intervals crossing zero. Although the methods used were adjusted for baseline differences, the control arm was found to have a lower mean baseline utility score and a higher mean follow-up utility score when compared to the intervention arm, it is therefore reasonable to suggest that the small incremental QALY gain for the intervention arm was due to underlying baseline differences. This lack of clear effect translates into pervasive uncertainty within the cost-effectiveness results even at high threshold willingness to pay values for a QALY gain. This is reflected in the cost-per childhood obesity case prevented (cost-effectiveness analysis) whereby the control dominated the intervention arm.

Compared to the amount of evidence available for the clinical effectiveness of obesity intervention, there have been few reported economic evaluations. Between January 2001 and April 2017, seven economic evaluations of school-based obesity interventions were published [30–36]. In all of these a cost-effectiveness analysis was applied measuring intervention effect using weight-specific outcomes, for example BMI or waist circumference. These studies all reported cost-effectiveness as a ‘cost per weight-specific outcome’. Without a commonly accepted threshold value for a unit gain in outcome, it is difficult to judge value for money. More recently, the results of the Healthy Lifestyles Programme (HeLP) study, a similar school-based obesity prevention trial in the UK run concurrently to the WAVES trial, was published [37]. In this trial the intervention incurred a small cost, however, there was not enough evidence of an effect of the intervention on preventing overweight or obesity. The evidence from the HELP study, alongside this WAVES trial, suggests that obesity prevention interventions delivered through schools are not enough in isolation to prevent childhood obesity. These findings provide support for a more comprehensive strategy to tackle the obesity epidemic, understanding the wide range of determinants of obesity, and the complex interaction of the school, family and social and physical environment as risk factors for obesity.

There are strengths to this study. The WAVES trial is one of the largest childhood obesity prevention trials undertaken to date within a socioeconomically and ethnically diverse population. The intervention was designed using a phased approach guided by the Medical Research Council framework for complex interventions [38]and contained elements pertaining to healthy eating and physical activity, and incorporated a range of behaviour change techniques. The economic evaluation conducted alongside used utility instruments designed for use in children, and a bottom-up micro-costing approach to assess the costs of the intervention.

In terms of limitations, there was a large amount of missing data on the resources used by teaching staff. School staff were asked to complete log books to record resource use, however given the multi-faceted nature of the intervention and the time burden of completing these log books, obtaining this data proved difficult. Other data was missing due to consented children being absent on the day of measurements, children not agreeing to being measured on the day, or children moving to other schools and parents withdrawing consent between baseline and follow-up. Consequently, we had to rely on multiple imputation and assume that data was missing at random. Similarly, due to available software limitations, it was not possible to include costs and outcomes within the same imputation equation, so there may be associated bias. To conduct the analyses we assumed there were zero costs to the control arm, and that any costs associated with the intervention arm were purely additive, i.e. in addition to ‘usual activities’, however the WAVES intervention might of substituted some of the school’s existing policy on healthy eating/exercising behaviours. Also, this study was not designed to capture costs associated with contacts with the health service and therefore the perspective for the cost analysis was purely from the school perspective. Had the intervention shown an effect on childhood obesity then any future health care cost-savings associated with preventing obesity over the long-term would have been captured. Finally, there remains an ongoing methodological debate concerning the use of the CHU-9D to capture health-related quality of life in school children and whether the instrument is responsive to change in weight status [39].

At first glance the results of this economic evaluation appear to show the WAVES intervention to be a cost-effective use of public resources, however there are high levels of uncertainty evidenced by the low probability of cost effectiveness at varying threshold levels of willingness to pay. This study therefore provides further support for a childhood obesity strategy that is comprehensive and goes beyond the school environment to consider the upstream determinants of obesity, as well as the wider social, physical and family environment. It provides evidence that schools can play a positive role towards tackling childhood obesity but that this needs to be part of a comprehensive multisectoral approach.

## Supporting information

S1 Table Resource use: WAVES intervention set up and development. (DOCX)

S2 Table Missing health utility data. (DOCX)

S3 Table Missing resource use data. (DOCX)
