# Supplementary material for: Economic evaluation of a childhood obesity prevention programme for children: Results from the WAVES cluster randomised controlled trial conducted in schools
Source: PLoS One. 2019 Jul 10;14(7):e0219500. doi: 10.1371/journal.pone.0219500 (PMC6619792; doi:10.1371/journal.pone.0219500)
Supplement: S1 Table — (DOCX) [file pone.0219500.s001.docx]

**S1 Table. Resource use: WAVES intervention set up and development**

| **Component** | **Resource Type** | **Resource use per class (SD)** | **Mean cost per class, £ (SE)** |
| --- | --- | --- | --- |
| Intervention handbook development | *Staff time (hours):*  Professor  Senior research fellow  Research fellow  *Printing handbooks:*  Number of handbooks | 0.025  0.025  0.15  2.5 | 1.11  0.81  2.78  11.47 |
| Intervention set-up meeting (researcher visit to school) | *Staff time (hours):*  Research fellow travel/meeting time  Teacher meeting time  *Travel cost (miles):*  Mileage | 1.014 (0.647)  0.324 (0.133)  18.6 (19.39) | 18.79 (1.896)  6.53 (0.423)  8.37 (1.38) |
| Development of cooking workshop/classroom materials | *Staff time (hours)*  Research associate  Research fellow | 6.5  1.75 | 107.12  32.45 |
| Preparing trainers for central training session | *Staff time (hours)*  Research associate  Research fellow | 0.325  0.2 | 5.36  3.71 |
| Adapting Villa Vitality for children aged 6-7 years | *Staff time (hours)*  Senior research fellow  Research fellow  Research associate | 0.125  0.25  0.25 | 4.06  4.64  4.12 |
| Preparing Villa Vitality teacher packs and liaising with schools | *Staff time (hours)*  Research associate | 1.5 | 24.72 |
| Total mean set up/development costs per school (£)  Total mean set up/development costs per class (£)  Total mean set up/development costs per consented child (£)  Total mean set up/development costs per intervention child, assuming class size =30 children (£) |  |  | 363.14  236.04  13.70  7.87 |
