# Supplementary material for: Economic evaluation of a childhood obesity prevention programme for children: Results from the WAVES cluster randomised controlled trial conducted in schools
Source: PLoS One. 2019 Jul 10;14(7):e0219500. doi: 10.1371/journal.pone.0219500 (PMC6619792; doi:10.1371/journal.pone.0219500)
Supplement: S2 Table — (DOCX) [file pone.0219500.s002.docx]

**S2 Table. Missing health utility data**

| **Time Point** | **CHU-9D completed (n (%))** | **Percent missing: control arm** | **Percent missing: intervention arm** |
| --- | --- | --- | --- |
| **Baseline** | 1350 (92%) | 7% | 9% |
| **Follow up one** | 1215 (83%) | 20% | 15% |
| **Follow up two** | 1131 (77%) | 25% | 21% |
