# Supplementary material for: Economic evaluation of a childhood obesity prevention programme for children: Results from the WAVES cluster randomised controlled trial conducted in schools
Source: PLoS One. 2019 Jul 10;14(7):e0219500. doi: 10.1371/journal.pone.0219500 (PMC6619792; doi:10.1371/journal.pone.0219500)
Supplement: S3 Table — (DOCX) [file pone.0219500.s003.docx]

**S3 Table. Missing resource use data**

| *Intervention cost sub-component* | *Missing data (%)* |
| --- | --- |
| Intervention handbook development & set up | 0 (collected by trial team) |
| Development and preparation for CW central training | 0 (collected by trial team) |
| Adapting VV materials and developing VV packs | 0 (collected by trial team) |
| Cooking workshop central training | 26% |
| Development of signposting materials | 0 (collected by trial team) |
| Signposting printing and postage | 0 (collected by trial team) |
| Physical activity and Villa Vitality package costs | 0 (collected by trial team) |
| Physical activity term 1 | 55% |
| Physical activity term 2 | 45% |
| Physical activity term 3 | 70% |
| Cooking workshop & classes - breakfast | 52.5% |
| Cooking workshop & classes - lunch | 57.5% |
| Cooking workshop & classes - dinner | 75% |
| Villa Vitality day 1 | 40% |
| Villa Vitality class project & challenges | 47.5% |
| Villa-Vitality – school visit | 45% |
| Villa-Vitality day 2 | 40% |
